# Study on effect of salinity level of water on electrocardiogram and some of blood serum minerals in grass carp, *Ctenopharyngodon idella*

**Published:** 2013

**Authors:** Ala Enayati, Rahim Peyghan, Ahmad Ali Papahn, Gholam-Hosain Khadjeh

**Affiliations:** 1 *Department of Clinical Sciences, Faculty of Veterinary Medicine, Shahid Chamran University of Ahvaz, Ahvaz, Iran; *; 2 * Department of Basic Sciences, Faculty of Veterinary Medicine, Shahid Chamran University of Ahvaz, Ahvaz, Iran.*

**Keywords:** Electrocardiogram, Grass carp, Minerals, Salinity

## Abstract

In this study the effects of salinity on the electrocardiogram and some of blood serum minerals in grass carp were investigated. For this purpose, grass carp were exposed to concentrations of 4, 8 and 12 g L^-1^ salinities and electrocardiogram of all fish was recorded. Blood samples were also collected from all fish and serum minerals were measured. Our results indicated that mineral level in the studied groups was significantly different. The average of heart rate per minute in control group and 4, 8 and 12 g L^-1^ were 10.15, 10.06, 12.17 and 7.79 beat per min, respectively. In 8 g L^-1^ group only the average of QT and ST segment decreased significantly in comparison with the control group (*p *< 0.05). In 12 g L^-1^ group the average of heart rate per minute decreased significantly in comparison with 8 g L^-1^ group (*p *< 0.05) and no difference in the average of heart rate per minute was observed in all groups. The average of RR, PT and ST segment in the 12 g L^-1^ group were significantly more than the other groups (*p *< 0.05)*.* The results showed that water salinity level increased to concentrations above 8 g L^-1^ can have significant effect on the electrocardiogram and mineral level of blood serum in grass carp. These changes are probably can be considered as one of the causes of impairment of health and death of this fish species in the salinities more than 8 g L^-1^ due to ion imbalance and cardiovascular disorders.

## Introduction

Any environmental disturbance such as salinity changes can be considered as a potential factor in changing hemo-stasis as it prompts a many responses in fish to deal with the physiological changes. These responses in fish can be detected in the form of changes in mineral element concentrations in the blood or alteration in heart activities. Considering that in some parts of Khuzestan province water salinity of fish ponds may be reached to concentrations higher than usual in some months of the year, warrants the need to study the effects of higher salinity on aquatic animals. In some situations it may accidentally or forced to use water with salinity higher than fresh water in fish farming. Some regions of the country (i.e. Yazd), has brackish water (with salinities higher than 2 g L^-1^). Also in recent years, due to the phenomenon of drought and reduced rainfall in Khuzestan province, water salinity levels in some fish farms increased to concentration of 9 g L^-1^. Therefore, the need to study the adverse effects of salinity on farmed fish seems to be necessary. In this study grass carp as one of the most important fishes of aquaculture industry in Khuzestan and also as a model to determine salinity effects on serum minerals and electrocardiogram of other fishes have been used. 

The electrocardiogram (ECG) is considered a simple and useful way to examine the physiological status of the heart in fishes and it has been used to evaluate the response of fishes to environmental changes.^1^ Labat was the first to record the ECG in the aquatic animals which studied the effect of temperature, salinity and light and some other factors in fish.^2^ In the following years, additional research on factors evaluating and changes in environmental conditions of fish and their impact on heart rhythm and activity using this method were carried out. However, little information is available about the electrocardiogram of fish. Many studies have been carried out on the fish heart functions by the measurement of ECG. They found various cardiac responses against different conditions, in terms of, fish species and environmental conditions.^3^^-^^5^

Teleost fish are able to regulate the ionic concentration of the internal body fluids within narrow ranges. Many studies reveal that exposure of freshwater fish to higher salinities, can change the internal osmolarity of plasma allowing an influx of water.^6^^-^^9^

Many intrinsic and extrinsic factors interact to influence HRs and patterns of cardiovascular function in fish. This function is set by the balance between these two sets of interacting intrinsic and extrinsic factors. The most important minerals that can affect cardiac activity are Na, K, Ca, P ions. In this study, electrocardiogram and the four main ions were used as an indicator of heart activity. The present study examines the possible effects that salinity level and osmo-regulatory processes can have on the blood serum minerals levels and electrocardiogram of grass carp that are subjected to gradually increasing level of environmental salinity.

## Materials and Methods

**Fish and experimental groups. **In this study 120 grass carp (weighting 100 to 120 g) were collected from earth ponds and transferred to the aquariums (12 aquariums, 150 liter each). For adaptation, fish were placed for a week in aquarium containing decolorized tap water at 19-21 °C. The fish were divided randomly in to 4 groups (10 fish in each group and three replicates per each treatment). Three groups were held in 3 different salinities at concentrations of 4, 8 and 12 g L^-1^. The forth group was reared in fresh water and considered as the control. For increasing water salinities in experimental groups, natural sea salt was added to the water. During the study period, twice a day the fish was fed by plated food (Feeding rate: 2% of fish body weight). The water was aerated properly to supply oxygen to water at level near the saturation level. During the test, physicochemical properties of water such as temperature and salinity were measured daily by salinity meter. The gradual increase of salinity in experimental groups was done in 3-4 days.

**Sampling and measurements.** After 3 weeks, the fish were caught and electrocardiogram of all fish was recorded.^3^ For recording the electrocardiogram, electro-cardiograph (Model FX-1201, Cardimax, Tokyo, Japan) was set in 25 mm per sec speed and voltage of 2 mV equal to 1 cm. At first fish were anesthetized by 100 ppm concentration of MS_222_, Then, electrocardiograms were recorded by using limb bipolar lead I. 

Blood samples were also collected from all fish. For blood collecting, bleeding was performed from the caudal peduncle vein. Blood samples were immediately transferred to sterile tubes and the serum was separated by centrifugation and levels of important blood serum minerals (sodium, potassium, calcium, phosphorus) were measured. In this study, calcium and phosphorus were measured manually using conventional biochemistry laboratory kits and spectro-photometry. Sodium and potassium were measured by the flame photometer (Model PFP7, Jenway, Essex, UK).

**Statistical analysis. **After blood analysis and recording of the electrocardiogram of all fish, means of each parameter and changes including: heart rate, time intervals (ST, PT, QR, QT, RR, PR segments) and serum minerals were measured and compared in all groups. The data were analyzed by SPSS (version 18, SPSS Inc., Chicago, IL, USA) and at significant level of *p* > 0.05 was compared by one way analysis of variance.

## Results

**Fig. 1:**
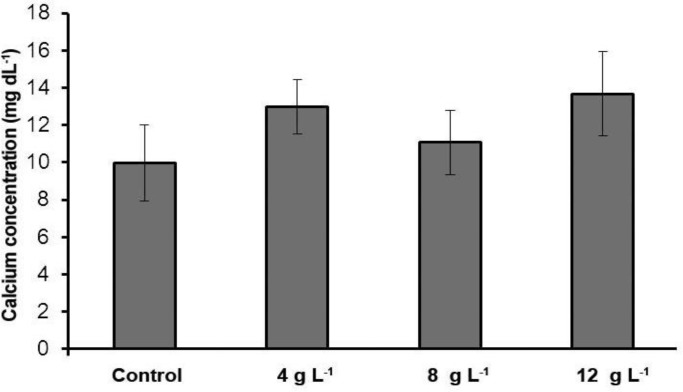
Serum calcium concentration of different experimental groups and the control (Mean ± SD).

**Fig. 2:**
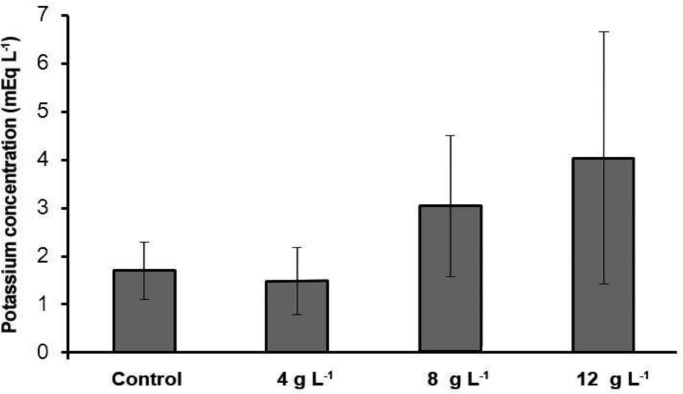
Serum potassium concentration of different experimental groups and the control (Mean ± SD).

**Fig. 3:**
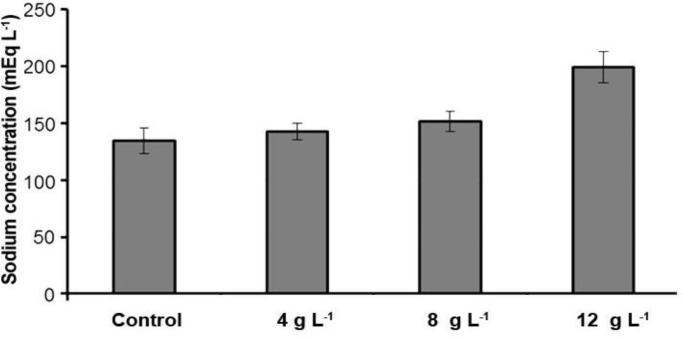
Serum sodium concentration of different experimental groups and the control (Mean ± SD).

**Fig. 4:**
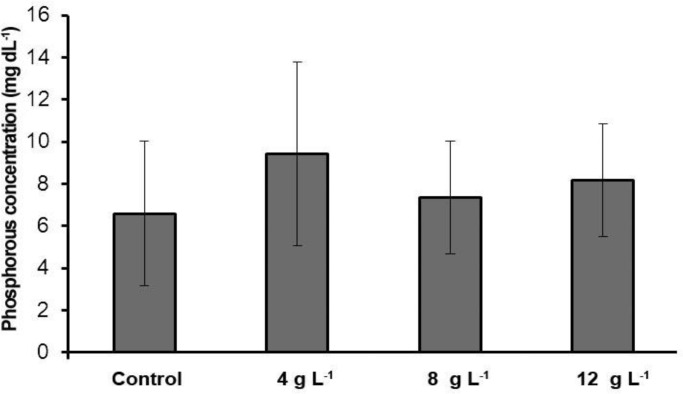
Serum phosphorous concentration of different experimental groups and the control (Mean ± SD).

**Blood serum minerals. **Our results indicated that sodium and calcium level in 4 g L^-1^ group salinity was significantly higher than the control group (*p *< 0.05) (Table 1). However, potassium levels in comparison with the control group had no significant difference (*p* > 0.05). In this study, sodium and potassium level in the 8 g L^-1^ group salinity increased significantly in comparison with the control group but the calcium and phosphorus levels in this group in comparison with the control group had no significant difference (*p *> 0.05)*.* Sodium, potassium and calcium levels in the 12 g L^-1^ group salinity in comparison with the control group had significant increase (Figs. 1, 2, 3 and 4) (*p *< 0.05) but the phosphorus level in this group in comparison with the control group had no significant difference (*p *> 0.05)*.*

**Table 1:** Some of blood serum minerals in grass carp in experimental groups and the control (Mean ± SD).

Group	**Parameters**			
	**Sodium ** **(mEq L** ^-1^ **)**	**Potassium ** **(mEq L** ^-1^ **)**	**Calcium** **(mg dL**^-1^**)**	**Phosphorus (mg dL** ^-1^ **)**
**Control**	ᵅ134 / 28 ± 11 / 37696134.28 ± 11.37 ^а^*	1 / 7014 ± 0 / 59448 1.70 ± 0.59 ^b^	9 / 9920 ± 2 / 03650 9.99 ± 2.03 ^b^	6 / 5936 ± 3 / 42018 6.59 ± 3.42 ^a^
**4 (g L** **-1** **)**	142 / 67 ± 7 / 19508142.67 ± 7.19 ^b^	1 / 4987 ± 0 / 69165 1.49 ± 0.69 ^b^	ᶜ13.00 ± 1.45 ^c^	9 / 4222 ± 4 / 37475 9.42 ± 4.37 ^b^
**8 (g L** **-1** **)**	151 / 15 ± 9 / 00042151.15 ± 9.00 ^c^	3 / 0216 ± 1 / 46103 3.02 ± 1.46 ^a^	11 / 0754 ± 1 / 72222 11.07 ± 1.72 ^b^	7 / 3720 ± 2 / 68096 7.37 ± 2.68 ^ab^
**12 (g L** **-1** **)**	198 / 89 ± 13 / 77153 198.89 ± 13.77 ^d^	4 / 0389 ± 2 / 61448 4.03 ± 2.61 ^a^	13 / 6752 ± 2 / 25274 13.67 ± 2.25 ^ac^	8 / 1711 ± 2 / 68786 8.17 ± 2.68 ^ab^

* Different letter in each column showed significant difference (*p *< 0.05)*.*

**ECG parameters. **According to our results, the average of heart rate per minute in the control group and in 4, 8 and 12 g L^-1^ salinity were 10.15, 10.06, 12.17 and 7.79 respectively (Table 2). The average of heart rate per minute and also average distance from the ECG in 4 g L^-1^ salinity in comparison with the control group had no significant difference (*p *> 0.05)*.* In 8 g L^-1^ group salinity, only the average of QT and ST segment decreased significantly in comparison with the control group (*p *< 0.05)*.* In 12 g L^-1^ salinity the average of heart rate per minute decreased significantly in comparison with 8 g L^-1^ salinity group (*p *< 0.05) and no difference in the average of heart rate per minute was observed in other groups. The average of RR, PT and ST segment in the 12 g L^-1^ salinity were more than other three groups significantly (*p *< 0.05). In 12 g L^-1^ group salinity, the average of QR segment decreased significantly in comparison with control and 4 g L^-1^ salinity groups (*p *< 0.05)*,* also in this group the average of QT segment increased significantly in comparison with the 4 and 8 g L^-1^ salinity groups (*p *< 0.05)*.*

**Table 2:** Some of electrocardiogram parameters in grass carp in experimental groups and the control (Mean ± SD).

Groups	Parameters						
	Heart rate (bpm)	RR interval RR(sec)	PRPR interval (sec)	STST interval (sec)	PTPT interval (sec)	QR interval QR (sec)	QT interval (sec)
Control	ᵅᵇ 10.± 1580/1010 ±76328/3 3.70 ab*	6.7564/6 ± 70 ±62068/2 2.60 b	ᵅ0.10 ± 03303/00.03 a	5612/0 ± 0.50 ± 10529/00.10 b	6860/0 ±0.60 ±12527/0 0.10 b	0352/0 ± 0.03 ±00770/0 0.00 b	6124/0 ± 0.60 ±10541/0 0.10 ac
4 (g L-1 )	ᵅᵇ10.00 ±62955/4 4.60 ab	9514/6 ± 6.90 ± 260495/2.60 b	1373/0 ± 0.10 ± 03169/00.03 a	5242/0 ± 0.50 ± 12176/00.10 bc	6681/0 ±0.60 ±10929/0 0.10 b	0354/0 ± 0.03 ±00859/0 0.00 b	5650/0 ± 0.50 ±11752/0 0.10 bc
8 (g L-1 )	1765/12 ± 12.10 ±43129/5 5.40 b	6507/5 ± 5.60 ± 90851/11.90 b	1339/0 ± 0.10 ± 03102/00.03 a	4745/00.40 ±±10966/ 0 0.10 c	6233/0 ±0.60 ±11768/0 0.10 b	0303/0 ± 0.03 ±01185/0 0.01 ab	5239/0 ± 0.50 ±11161/0 0.10 b
12 (g L-1 )	77958/7 ±.70 ±05768/4 4.00 a	6575/9 ± 9.60 ± 44013/44.40 a	1242/0 0.10 ± 4333/ 0±0.40 a	6492/0 ± 0.60 ± 13296/00.10 a	7971/0 ±0.70 ± 15267/00.10 a	0267/0 ± 0.02 ±01341/0 0.01 a	6996/0 ± 0.60 ±14275/0 0.10 a

* Different letter in each column showed significant difference (*p *< 0.05); bpm = beat per min.

## Discussion

Biochemical evaluation of serum is considered as part of the diagnostic method of a patient fish. In fact most of blood biochemical studies of fish have been devoted to economically important fishes such as Salmonidae (salmon and trout), catfish and cyprinids.^6^ Optimal salinities for growth and metabolic rates were influenced by species and life stage.^8^ In our study we observed that with increasing salinity at concentrations of 4, 8 and 12 g L^-1^, serum sodium level in these three groups were significantly higher than the control group. Sodium, potassium and calcium level in the 8 and 12 g L^-1^ groups increased significantly in comparison with 4 g L^-1^ salinity and the control groups. The phosphorus level in the group with 4 g L^-1^ salinity is significantly higher than the control group. The results showed an increase in water salinity can have a significant impact on blood serum minerals. These changes have been severe especially in the higher salinity of 8 g L^-1^. Therefore, these changes can be effective in the pathogenesis of salinity stress condition, osmotic imbalance and fish death.

In culture conditions, adult carp usually can survive successfully in brackish water with salinities of up to 9 ppt. Bronchial chloride cell numbers decreased after brackish water exposure, whereas a gradual increase was observed in chloride cell size.^10^^,^^11^ Increased salinity was also recorded to result in many more chloride cells in the gills tissue.^11^ Sampaio and Bianchini investigated salinity effects on osmo-regulation and growth of the euryhaline flounder *Paralichthys orbignyanus*, results suggest that the lower growth rate exhibited by *P. orbignyanus* in freshwater could be due, at least partially, to a higher energy expenditure associated to a higher branchial Na^+^, K^+^ -ATPase activity in this environment.^8^ Most studies that involve exposing anadromous salmonid fishes to seawater report an increase in gill Na^+^/K^+^-ATPase activity suggesting that it is an integral part of their successful acclimation.^12^ Salinity and its variations are among the key factors that affect survival, metabolism and distribution during the fish development.^13^ Teleost fish are able to regulate the ionic concentration of the internal body fluids within narrow Ranges. The data reveal that upon exposure to 1% (w/w) NaCl, the internal osmolarity (plasma) of the carp attained values which were 30-45% higher than the external, allowing an influx of water.^7^ Reduced osmoregulatory abilities and reductions in maximal swimming performance suggest that high salinity may constrain activity and salinity adaptation in euryhaline fishes should take into account the interactive effects of salinity on physiology and behavior.^14^

Many investigations on effect of various factors on ECG of fish have been done; however, few studies have reported the effect of salinity on fish electrocardiogram.^5^^,^^14^

Since ECG recording in fish is not an ordinary procedure, the ECG records of fish are scarce. It is difficult to eliminate electric noise in actively swimming fish while recording ECGs. In the present study, we attached electrodes to three points on the ventral surface of anesthetized fish and obtained data successfully.

In the early and extensive work of Labat, some electro-cardiogram features were related to ecological factors.^2^ Hypoxia could cause an increase of amplitude in T wave and a decrease of P wave, and high temperatures showed a decrease of the PR interval and even fusion of T with the next P wave. Other factors like environmental pressure, light, salinity were also assessed in this work as influencing the heart signal.^2^ Salinity stress in the stenohaline fish species may cause rapid and extreme modifications of heart beat or tachycardia followed by a bradycardia and ultimately fish death.^15^ According to result, the average of heart rate per minute in control group and groups with 4, 8 and 12 g L^-1^ salinity were 10.15, 10.06, 12.17 and 7.79 respectively. The average of heart rate per minute and also average of distance in the ECG in the 4 g L^-1^ group salinity in comparison with the control group had no significant difference. In the 8 g L^-1^ salinity group, only the average of QT and ST segment decreased significantly in comparison with the control group (*p *< 0.05). But in the 12 g L^-1^ group, the average of heart rate per minute decreased significantly in comparison with the other groups and the control. Our results of electrocardiogram parameters showed that an increase of water salinity more than 8 g L^-1^ can have a significant impact on the ECG. Therefore one of the causes of death in the higher salinity can be due to cardiovascular disorders. In experimental groups all parts and segments of the electrocardiograms curves were present and no parts was missing, so we conclude that this type of bradycardia and tachycardia observed in different salinities, derived from effect on SA node. Therefore, we can attribute it as sinusoidal bradycardia and tachycardia. 

In this study was found that RR intervals were not the same in all records. It was found in all fishes (the control group and the experimental groups) which represented a type of arrhythmia (sinus arrhythmia). Other studies showed that sinus arrhythmia can be considered normal in fish.^6^ The bradycardia observed in this study could be due to the increased potassium. Increasing potassium in extracellular fluid may cause heart dilation and decrease in heart rate. Decrease in heart bit may limit the oxygen uptake through the gills, because the oxygen uptake is related to the amount of counter-current between the water flowing through the gill lamellae and the blood circulation of the gill. In fingerling grass carp that were acclimated to experimental salinities of more than 1 ppt, it was showed that oxygen consumption rates of fish declined, as salinity increased.^16^ Also decrease of pH in the blood has been confirmed as the cause of anoxia in fish exposed to stress.^16^ As osmo-regulatory costs are linked to metabolic activity through ventilation, the portion of energy necessary to compensate for the ventilation related osmotic and ionic loss will increase as fish metabolic expenditure rise.^17^

We consider that the interaction of many causes mentioned above resulted in the death of the fish in higher salinities. It has been known that the heart rate of swimming fish increases within a restricted range. Therefore, the heart rate was expected to increase in the struggling fish at the beginning of the exposure to salinity. According to Kojima *et al.* heart rate slightly decreased from midnight to dawn, so they conclude that the metabolic rate of cultured fish is lower during the nighttime than in the daytime.^14^

Development and growth in fish are controlled by internal factors including CNS, endocrinological and neuroendocrinological systems. Among vertebrates, they are also highly dependent on environmental conditions. Among other factors, many studies have reported an influence of water salinity on fish development and growth.^18^^-^^22^ Any behavioral or environmental change that alters oxygen uptake typically involves a change in cardiac output. In conclusion the results of electrocardiogram parameters and serum minerals changes showed that increase of water salinity more than 8 g L^-1^ can have a significant impact on the ECG and minerals.
